# Antirelapse Efficacy of Various Primaquine Regimens for *Plasmodium vivax*

**DOI:** 10.1155/2014/347018

**Published:** 2014-09-10

**Authors:** D. D. Rajgor, N. J. Gogtay, V. S. Kadam, M. M. Kocharekar, M. S. Parulekar, S. S. Dalvi, A. B. Vaidya, N. A. Kshirsagar

**Affiliations:** ^1^Department of Clinical Pharmacology, Seth G.S. Medical College and KEM Hospital, Parel, Mumbai 400 012, India; ^2^Microbiology and Immunology, Center for Molecular Parasitology, Drexel University College of Medicine (DUCM), Philadelphia, PA 19104, USA; ^3^National Chair Clinical Pharmacology, Indian Council of Medical Research (ICMR), Government of India, New Delhi 110 002, India; ^4^Employee's State Insurance-Post Graduate Institute of Medical Sciences & Research (ESI-PGIMSR), Government of India, Mahatma Gandhi Memorial (MGM) Hospital, Dr. S.S. Rao Road, Parel, Mumbai 400 012, India

## Abstract

*Background*. Efficacy of standard dose of primaquine (PQ) as antirelapse for *P. vivax* has decreased. We aimed to assess efficacy of different PQ regimens. *Methods*. It was an open label, randomized, controlled, parallel group, assessor blind study comparing antirelapse efficacy of 3 PQ regimens (B = 15 mg/day × 14 days, C = 30 mg/day × 7 days, and D = 30 mg/day × 14 days) with no PQ group (A) in *P. vivax* patients. Paired primary and recurrence samples were subjected to 3 methods: (i) month of recurrence and genotyping, (ii) by PCR-RFLP, and (iii) PCR sequencing, to differentiate relapse and reinfection. The rates of recurrence relapse and reinfection were compared. Methods were compared for concordance between them. *Results*. The recurrence rate was 16.39%, 8.07%, 10.07%, and 6.62% in groups A, B, C, and D, respectively (*P* = 0.004). The relapse rate was 6.89%, 1.55%, 4%, and 3.85% as per the month of recurrence; 8.2%, 2%, 4.58%, and 3.68% (*P* = 0.007) as per PCR-RFLP; and 2.73%, 1.47%, 1.55%, and 1.53% as per PCR sequencing for groups A, B, C, and D, respectively. The concordance between methods was low, 45%. *Conclusion*. The higher recurrence rate in no PQ as compared to PQ groups documents PQ antirelapse activity. Regimens tested were safe. However, probable resistance to PQ warrants continuous monitoring and low concordance and limitations in the methods warrant caution in interpreting.

## 1. Introduction

Malaria continues to be a major global health problem exposing over 2000 million of the world's population to varying degrees of malaria risk. Of the four parasite species,* P. falciparum*,* P. vivax*,* P. malariae,* and* P. ovale* that cause malaria,* P. falciparum* and* P. vivax* are the two major parasite species seen in the country, India. It is estimated that 1.2–1.5 million new cases of malaria occur in India each year [1], 65% caused by* P. vivax*. In the city of Mumbai,* P. vivax* accounts for 80% of the total malaria cases [2]. Despite this burden,* P. vivax* species remains less well studied.

Worldwide, the focus of research in malaria has centered around* P. falciparum* because of the development of resistance by this species to the standard antimalarial drugs and the mortality associated with the species. However,* P. vivax* is the most widely distributed parasite being the most common cause of malaria imported into many areas in which malaria is not endemic [3–5] with an increasing trend of severe and fatal cases and more importantly, the high morbidity associated with recurrence of infection caused by the relapses. Despite numerous studies carried out to evaluate antirelapse potential of existing and new antimalarials such as Tafenoquine, and its combinations [6–8], Primaquine (PQ), an 8-aminoquinoline, remains at present the only agent available worldwide to prevent relapses [9]. Unfortunately, there has been emergence of PQ nonresponsiveness in patients with* P. vivax* who received World Health Organization (WHO) recommended antirelapse regimen [10], 15 mg/day for 14 days of primaquine [11–14]. Despite the surge of resistance monitoring, the in vitro resistance testing has not achieved much success for* P. vivax*. Although protocols to culture the species ex vivo have been developed [15–17], they remain unsuited for routine assays. Thus, well-conducted clinical trials still remain the mainstay and an essential component of* P. vivax* resistance monitoring. However, in clinical trials, identification of relapses gets further confounded by the inability to distinguish relapses, that is, PQ treatment failures, from reinfections, that is, treatment successes which clinically or parasitologically manifest during the follow-up period on account of the infection with fresh mosquito bite. Even though distinguishing recrudescence from reinfection has been largely overcome for* P. falciparum* by use of genotyping methods [18], the application of the genotyping methods in case of vivax is not much explored.

Thus, the primary objective of the present clinical study was to assess the efficacy of various PQ regimens as antirelapse treatment along with no PQ regimen for* P. vivax* malaria patients in Mumbai, India, by monitoring the recurrence rate as well as distinguishing the recurrence as relapse or reinfection using three different methods. The secondary objectives of the study were to monitor the safety and find the concordance between the results of the three methods used to differentiate relapse and reinfection and the diversity of the types of strains prevalent in the samples of the study population.

## 2. Materials and Methods

### 2.1. Ethics

The Institutional Ethics Committee approval and patient's informed consent were obtained prior to enrollment in the study.

### 2.2. Study Design

It was an open label, randomized, controlled, parallel group, assessor blind study comparing antirelapse efficacy of 3 regimens of PQ, 15 mg/day × 14 days (group B), 30 mg/day × 7 days (group C), and 30 mg/day × 14 days (group D) along with a group of* P. vivax* patients who did not receive PQ (group A).

### 2.3. Participants

Patients suspected to be suffering from malaria were referred to Malaria Out-Patient Department (OPD), for microscopic diagnosis of malaria using peripheral blood smear stained using Giemsa stain. Patients diagnosed as positive for* Plasmodium vivax *malaria, willing to participate in the study, and fulfilling inclusion exclusion criteria of the study were enrolled in the study.

### 2.4. Inclusion Criteria

Inclusion criteria were as follows:

- adult patients, male and female (equal or over the age of 18 years);
- peripheral blood smear diagnosis of* Plasmodium vivax*;
- willing to undergo hospitalization for the entire duration of primaquine treatment;
- willing to provide informed consent;
- willing to undergo investigations and come for regular followup;
- normal G6PD;
- hemoglobin greater or equal to 10 gm/dL.

### 2.5. Exclusion Criteria

Exclusion criteria were as follows:

- mixed infection with* Plasmodium falciparum*;
- pregnancy and lactation;
- evidence of significant hepatic, renal, or cardiac disease as diagnosed by history, clinical examination, and laboratory tests whenever necessary;
- any other condition which would interfere with patient's participation in the study or compliance with the treatment.

### 2.6. Interventions

All the patients enrolled in the study were given the standard WHO recommended treatment regimen of Chloroquine (CQ) [19]. The treatment was initiated on day 1 with 10 mg/kg of CQ. Subsequently, patient received 10 mg/kg and 5 mg/kg of CQ on days 2 and 3, respectively. On day 4, as per the randomization, they received one of the three PQ regimens: 15 mg/day × 14 days (B), 30 mg/day × 7 days (C), or 30 mg/day × 14 days (D). The patients who were not eligible to receive PQ for a number of reasons such as Hg < 10 gm/dL, G6PD deficiency, pregnancy, and lactation formed the no PQ group (A).

### 2.7. Outcomes

The primary efficacy outcome of the study was the number of patients showing recurrence, relapse, and reinfection of* P. vivax* infection. The secondary outcome, safety, was assessed by monitoring the adverse events observed in patients. The adverse event evaluation was carried out by clinicians by monitoring the adverse events (symptoms, signs) that were not present at baseline or worsened during the study. The secondary outcome also included comparison of number of patients classified as relapse and reinfection by the three methods to find out the concordance between the methods used and the genetic diversity observed based on PCR sequencing method.

The recurrence was monitored by evaluating the efficacy of the CQ and PQ. CQ response was monitored as per the criteria given by Wernsdorfer et al. [20].

The PQ effect was assessed by regular followup once in a month, after the first 28 days for CQ sensitivity monitoring, by examination of peripheral blood smear until next 6 months. The peripheral smear examination was also done whenever patients developed fever within the 6-month follow-up period. Patients showing reappearance of* P. vivax* parasitemia between 1 to 6 months were considered as cases of recurrence. The cases of recurrence were classified as relapse or reinfection based on the three methods, the month of recurrence, and the two genotyping methods: PCR-RFLP and PCR sequencing.

#### 2.7.1. As per the Month of Recurrence

The recurrence was classified as relapse if it occurred in the period between January and June (low transmission season) and reinfection if it occurred in the period between July and December (active transmission season) [21].

#### 2.7.2. As per the Method of Genotyping

*(1) PCR-RFLP.* This method of genotyping involved use of* P. vivax* MSP3*α* (PvMSP3*α*) and MSP3*β* (PvMSP3*β*) as polymorphic regions. The protocols followed were adopted from the available literature [22, 23].

*(1.1) Interpretation.* The restriction enzyme (RE) banding patterns observed on the agarose gel for the paired samples were matched visually to classify them as relapse if it was the same and reinfection if it was different. The results of both the genes used (PvMSP3*α* or PvMSP3*β*) were pooled for the purposes of genotyping data analysis. When the RFLP pattern for PvMSP3*α* and PvMSP3*β* indicated contradictory results, meaning one showing same pattern while the other showing different RFLP pattern for the same paired sample, the parasite strains were considered as same strains thus categorizing the paired sample as relapse. The basis for this consideration was that it was unlikely to not see polymorphism, if present, for the polymorphic sites used. Thus the detection of same parasite with such a polymorphic region indicates presence of same parasite clones in the paired sample which means relapse. The presence of different clones as indicated with another RE probably points to either the presence of multiple clones in one of the samples or reactivation of one of the clones from primary infection [24]. 

*(2) PCR Sequencing*. This was carried out by selecting the polymorphic region of* P. vivax* MSP1 (PvMSP1) gene. The method was performed at one of the commercial laboratories, namely, Chromous Biotech Pvt. Ltd, Bangalore, India. The protocols followed were adopted from the available literature [25] with minor modifications. 

*(2.1) Sequencing of the Purified PCR Product. *The parasite DNA was amplified [25] and the PCR product was sequenced using ABI Big-Dye terminator cycle sequencing kit in ABI3100 sequencing machine. 

*(2.2) Sequence Analysis for Identification of Genotypes.* The raw sequence data obtained was coded. The amino acid sequences obtained from different samples were aligned using multiple alignment software (Clustal W). Based on the variation obtained, different genotypes were identified.

### 2.8. Sample Size

Our previous study has shown that patients not treated with PQ show the recurrence of* P. vivax* in 15–20% of patients [14, 26]. Also, the 15 mg/d × 14 days regimen of PQ is associated with 5% recurrence rate of vivax malaria of which 2% are true relapses. In the present study, it was to be assessed if 30 mg/day × 7 days and 30 mg/day × 14 days, which contain equivalent/higher dose of PQ, will have either equivalent (5%) or 0% recurrence rate. Therefore the sample size of 120 per group at 5% significance and 80% power of the study were initially considered adequate to detect the difference. However, eventually, we expected that the difference could be lower and also malaria being public health issue, we took larger sample size.

### 2.9. Randomization and Sequence Generation

A simple, computer generated randomization scheme was used for the randomization of patients into the three PQ regimen groups.

### 2.10. Allocation Concealment

This was an open label study and no concealment of treatment allocation was followed.

### 2.11. Implementation

**Figure 1:**
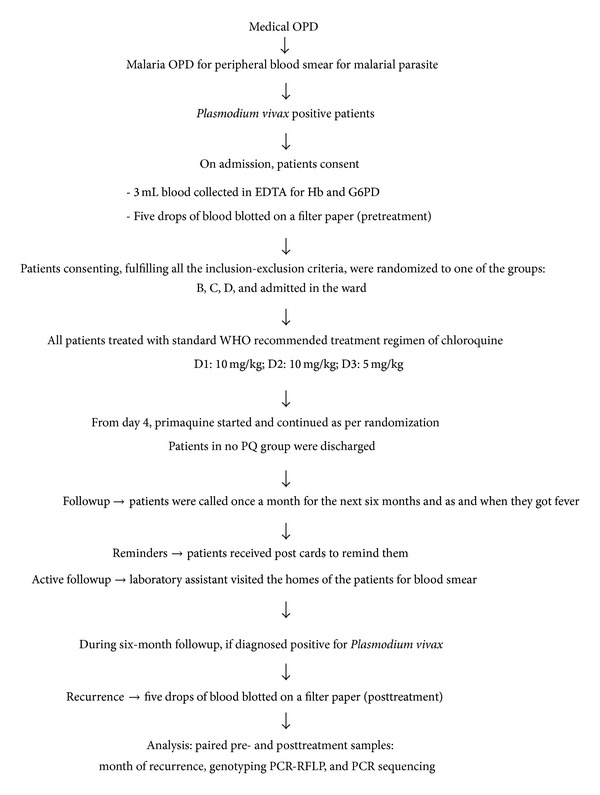
Flow chart of the study.

The patients fulfilling the inclusion exclusion criteria and consenting to participate in the study were enrolled in the study. The blood spots were blotted on the filter paper (pretreatment sample) for genotyping analysis. All the patients enrolled in the study were hospitalized either for 10 or 17 days depending on the PQ group they were randomized to and then treated with standard WHO recommended treatment regimen of CQ for 3 days, followed by PQ as per randomization. All the patients were given follow-up card at the time of discharge and were asked to follow up once a month for subsequent six months and also as when they get fever. In order to get maximum follow-up data and reduce drop outs due to lost to followup, post cards were sent to remind them about their follow-up visits. Active followup was also done when the laboratory assistant visited the homes of the patients and made blood smears, thus avoiding the need for the patient to visit the hospital. Any of these patients diagnosed to be positive for* P. vivax* during the period between 1 to 6 months of followup were considered cases of recurrence. These cases were readmitted and 3 mL of blood was collected again for hemoglobin measurement and five drops of blood were blotted on a filter paper (posttreatment sample). The paired pre- and posttreatment samples were subjected to genotyping analysis to distinguish recurrence as relapse or reinfection. The flow chart given at the end shows the schematic presentation of the plan of the study (Figure 1).

### 2.12. Blinding

Although the study was not blinded in terms of treatment administration, the person seeing the slides and carrying out other outcome assessments were blinded to the treatment group by coding the samples.

### 2.13. Statistical Methods and Data Analysis

Protocol method of analysis was used to analyze the data. Chi-square test was applied to find the differences between the 4 groups for baseline characteristics.

#### 2.13.1. Efficacy

The total number and percentages of patients showing recurrence of* P. vivax* malaria were expressed as a percent of the total number of patients who completed the 6-month followup of the study period. These percentages were compared amongst groups using the Chi-square or Fisher's test as appropriate. The number of paired samples available were subjected to classification of relapse and reinfection based on month of recurrence and genotyping (PCR-RFLP, PCR sequencing). Owing to the nonavailability of some of the paired samples for genotyping as well as nonamplification of some of the samples on account of the genotyping technique flaws, genotyping results were not available for all the samples. To compensate for the loss of data due to reasons listed, relapse and reinfection rates were extrapolated to total number of patients showing recurrence. This rate was then used for comparison.

The concordance between three methods was checked by comparing the results of the methods. The data of PCR sequencing were used to depict the diversity of different genotypes prevalent in the samples studied.

#### 2.13.2. Safety

The number of patients in each group who had adverse events or discontinued medication due to adverse events was expressed as a percent of the total. The percentages were compared using the Chi-square or Fisher's test as appropriate. For the purpose of the study, any adverse events (irrespective of cause-effect relationship) which were not present at baseline or worsened after baseline were taken for statistical analysis.

## 3. Results

### 3.1. Participant Flow and Recruitment Period

Overall 4215 (8.15%) cases of* P. vivax* were diagnosed in the total of 51,709 suspected cases screened, over a period from August 01 to Feberuary 04. Out of 4215 patients, 1556 patients were part of the study and 1,242 completed the 6-month followup, (group A = 305, group B = 322, group C = 298, and group D = 317). There was no difference in the baseline demographic characteristics of the study population (Table 1).

### 3.2. Evaluation of Efficacy

#### 3.2.1. Sensitivity to Chloroquine

All the 1556 patients (except 1 who was discontinued CQ due to AE) in 4 groups (A, B, C, and D) showed clearance of parasites by day 6 from the initiation of treatment, without subsequent reappearance until D28, showing that all* P. vivax* strains were sensitive to Chloroquine.

#### 3.2.2. Sensitivity to Primaquine

*(1) Recurrence*. There were a total of 127 patients who showed recurrence of* P. vivax* malaria, giving the cumulative recurrence incidence of 10% (127/1242). The number of patients showing recurrence of vivax parasitemia were 50 (16.39%), 26 (8.07%), 30 (10.07%), and 21 (6.62%) in groups A, B, C, and D, respectively. The rate of recurrence varied significantly in the 4 groups (*P* = 0.004). The number of patients showing recurrence was higher in patients who did not receive PQ as compared to those who received different regimens of PQ (Table 2).

*(2) Differentiation of Recurrence as Relapse or Reinfection*

*(2.1) As per the Month of Recurrence*. The relapse rate was 6.89%, 1.55%, 4%, and 3.85% and the reinfection rate was 9.51%, 6.52%, 6%, and 5.13% in groups A, B, C, and D, respectively. There was significant difference in the relapse rate in 4 groups (*P* = 0.009). The relapse rate was higher in no PQ group (6.89%) as compared to the relapse rate in three PQ groups (1.55%, 4%, and 3.85%) (Table 2).

*(2.2) As per the Method of Genotyping*.* (i) PCR-RFLP: PvMSP3α and PvMSP3β*. The relapse rate was 8.2%, 2%, 4.58%, and 3.68% and the reinfection rate was 8.2%, 5.82%, 5.49%, and 2.9% for groups A, B, C, and D, respectively. There was significant difference in the relapse rate in 4 groups (*P* = 0.007) (Table 2).  Figure 3 shows restriction enzyme digestion banding pattern used to differentiate relapse and reinfection.* (ii) PCR Sequencing: Pv MSP1*. The relapse rate was 2.73%, 1.47%, 1.55%, and 1.53% while the reinfection rate was 13.66%, 6.60%, 8.52%, and 5.09% in groups A, B, C, and D, respectively (Table 2).

### 3.3. Adverse Events

#### 3.3.1. Observed Adverse Events (AEs) of CQ

Out of 1556 patients, 9 (0.57%) patients (A = 1, B = 0, C = 4, and D = 4) reported adverse events during the CQ treatment. The number of AEs reported was significantly different in the 4 groups (*P* = 0.033) (Table 3). The AEs seen were itching (5), burning of palms (1), abdominal pain (1), loose motion (1), and heat boils (1). Of these 9 AEs, 1 AE of itching and mucous membrane on lips lead to discontinuation of CQ on D3.

#### 3.3.2. Observed Adverse Events of PQ

Out of 1556 patients, 31 (1.99%) patients (A = 3, B = 5, C = 10, and D = 13) reported adverse events during PQ treatment (after completion of CQ treatment). The number of AEs was significantly different in the 4 groups (*P* = 0.006) (Table 3). The AEs seen were nausea (1) acidity (02), weakness (1), itching (09), acidity plus itching (01), abdominal pain (04), acidity plus abdominal pain (01), epigastric pain (02), epigastric burning (01), weakness plus abdominal pain (01), vomiting (01), mucocutaneous lesions on left leg and gluteal region (01), boils on forehead and back with pus (01), boils rash-maculopapular-all over body (01), swelling of upper lips, legs, and palms with itching (01), severe pruritus (01), morbilliform rash in fingers (01), and boils inside the mouth and tongue (01).

Of these 31 patients, AEs in 11 patients (A = NA, B = 2, C = 2, and D = 7) lead to discontinuation of PQ treatment. The number of AEs leading to discontinuation of PQ treatment differed significantly in the 4 groups studied (*P* = 0.003) (Table 3). These AEs were itching (03), mucocutaneous lesions on the lower lip and gluteal region (01), formation of boils on forehead and back with pus (01), abdominal pain (01), acidity plus abdominal pain (01), morbilliform rash in fingers (01), epigastric pain (01), boils inside the mouth and tongue (01), and vomiting (01).

The number of drop outs due to AEs in 4 groups was significantly different (*P* = 0.001). The number of AEs was significantly higher in group D as compared to other groups (Table 3).

### 3.4. Concordance of the Three Methods of Classification Used

**Figure 2:**
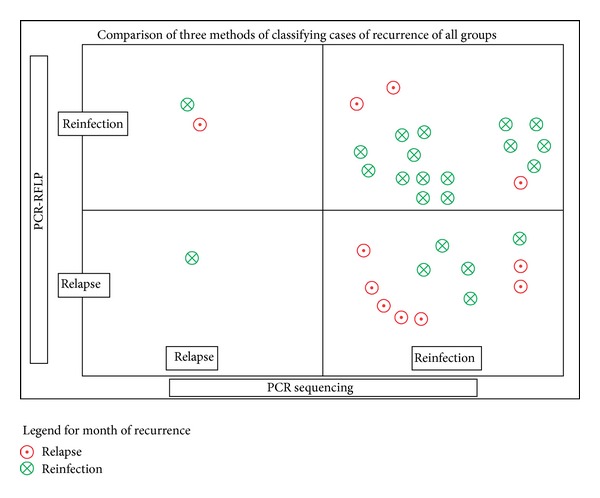
Comparison of three methods of classifying cases of recurrence: all groups.

**Figure 3:**
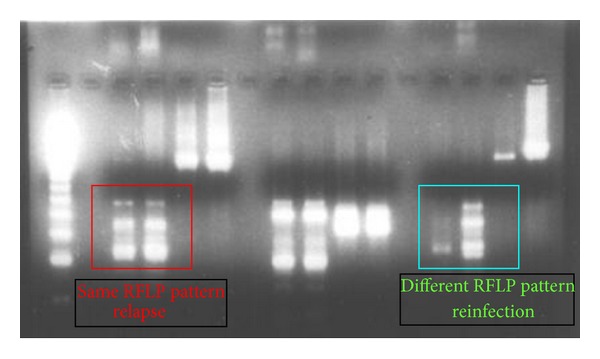
PCR-RFLP restriction enzyme digestion banding pattern.

There were 33 paired samples for which the results from all the three methods were available to assess the concordance. The concordance between all the three methods was 45% whereby all the three methods classified the case of recurrence as relapse or recurrence in the same way. While relatively high concordance was noted between PCR-RFLP and PCR sequencing (57.58%), good concordance was also noted between the month of recurrence and PCR-RFLP (48.48%) but little concordance between month of recurrence and PCR sequencing method (15.15%) (Table 4).  Figure 2 gives the overall concordance in the methods considering all groups together.

The rates of relapse and reinfection identified by the method of month of recurrence and PCR-RFLP were found to be in agreement. While PCR sequencing results were bit different than the results of both these methods of classification used (Table 2).

### 3.5. Genetic Diversity

Fifty-two genetic subtypes were identified in a total of 143 isolates (paired primary and recurrence samples) analyzed.

## 4. Discussion

The present comparative study carried out to assess safety and efficacy of different PQ regimens as antirelapse treatment identified a higher recurrence rate in the patients who did not receive PQ treatment as compared to the patients who received different PQ regimens. When the cases of recurrence were subjected to the methods differentiating relapse and reinfection, majority of them were found to be reinfection cases.

The study did not find any major or unexpected adverse events. However, significantly higher adverse events in higher dose of PQ, group D (30 mg × 14 days), leading to significantly higher drop outs were noted.

Discordance was noted in the results of the methods used to differentiate cases of recurrence as relapse or reinfection. The study also documented a lot of genetic diversity in the types of genotype identified using the PCR sequencing method of genotyping indicating the diversity of type of* P. vivax* strains prevalent in the population (geographic area) studied.

The low recurrence rate found in the patients receiving different PQ regimens as compared to patients not receiving PQ (16.3%) is indeed a good indicator of PQ effect and thus it is used as antirelapse treatment. The earlier study in the same population has documented recurrence rate of 9.2% in patients not treated with PQ [14]. The rates of recurrence as high as 40% in India [27] and 31% in Pakistan [13] have been documented in previous studies for patients not given PQ treatment.

The present study reported recurrence even in patients given PQ treatment. This also has been documented in earlier studies. For example, the study in the same population indicated recurrence rate of 4.6% for the 15 mg × 14 days PQ regimen [14]. This recurrence rate seems to have doubled over a period as found in the current study. Diminished effect of PQ, given in the standard dose of 15 mg × 14 days, has already been reported in earlier studies such as Smoak et al. [12] which reported recurrence rate as high as 43% in Somalia Army troops while Bunnag et al. [11] reported effectiveness of PQ to be only 82%. On the other hand, Leslie et al. [13] reported pretty low recurrence rates (1.8%) for this regimen.

In order to increase the effectiveness of PQ, use of higher dosages of PQ has been tried including a latest case report being successfully treated with high dose PQ as high as 45 mg once weekly for 8 weeks at 3rd instance; after the failure of 15 mg/d for 2 weeks at 1st instance and 7.5 mg 4 times daily for 2 weeks at 2nd instance for radical cure of vivax malaria [28]. Though there are not many studies assessing high dose PQ, a study administering higher PQ regimen did not show 100% effectiveness, reporting 89% cure rate for the 30 mg/day × 7 days PQ regimen [29] similar to the recurrence rate of 10.07% observed in the current study.

The recurrence of* P. vivax* at even higher dose PQ (30 mg/day for 14 days) has been documented earlier [30, 31].

Extreme variation is noted in the recurrence rates reported in these studies. Several factors including the type of prevalent genetic strains of vivax as well as the varied length of follow-up period used in the studies might explain the variations observed in these studies. The period of followup is an important consideration for observing the radical cure of* P. vivax*. With longer follow-up period, the chance of detecting more positive cases increases. Thus, the comparison of findings from different studies require due consideration of the follow-up period which might vary among studies. Also, the major limitation in assessing the effect of PQ as antirelapse is the confounding effect of reinfection in the area where malaria is endemic. Several of these earlier studies lack the data on PCR or genotyping corrected relapse rates.

The present study used 3 methods to differentiate the cases of recurrence as relapse or reinfection in order to better assess the antirelapse effect of PQ. It was noted that, although recurrence rates were high, when these cases of recurrence were classified as relapse or reinfection using different methods, relapse rates observed were much lower.

There is not much research involving genotyping of paired samples to compare with the present study. However, the PCR corrected rates of relapse observed in the current study were comparable to our earlier study [14] reporting the true relapse (PCR corrected) rate of 2% (PCR-SSCP) for 15 mg/day × 14 days as compared to 1.47% (PCR sequencing) and 2% (PCR-RFLP) noted in the present study.

The relapse and reinfection rates varied between the three methods used in the present study leading to discordance in the results of the three methods used to differentiate the cases of recurrence. There could be several reasons for this discordance ranging from the varying sensitivity and specificity of the method, the polymorphic markers used and certain unresolved issues like infection with multiple clones. For example, PCR sequencing technique could detect higher reinfection rate because it can differentiate the two isolates more precisely as compared to PCR-RFLP technique. The recent genotyping study done to find the genetic diversity of* P. vivax* in Kolkata, India, by Kim et al. [32], using three polymorphic markers (pvcs, pvmsp1, and pvmsp3*α*) concluded that genotyping protocols used by them may be useful for differentiating reinfection from relapse and recrudescence in studies assessing antimalarial drug efficacy. Two of these polymorphic markers were used in the present study. However, if the patient is getting infected with multiple clones, any method or test may not reliably differentiate relapse and reinfection. Thus differentiation of relapse and reinfection remains a major roadblock in assessing efficacy of antirelapse activity of antimalarials. This is also corroborated in an editorial by Collins [33]. In addition to the limitation of the methods itself, the study by Imwong et al. [24] has revealed heterologous reactivation of hypnozoites leading to recurrence, even in the patients from area where reinfection was ruled out. In such a case, observation of different genotypes in the recurrence sample does not indicate reinfection. If this is the case, then all efforts invested in differentiating the recurrence on the basis of genotyping technique seem to be in vain. This drawback of the technique, when mixed clones are present at the time of primary infection, has also been acknowledged previously by Kirchgatter and Del Portillo [34]. Probably, cloning experiments, which were not part of the current study, might provide some insight into the presence or absence of multiple clones in the given samples. It is also observed that the technique used for genotyping plays a very crucial role. PCR sequencing being the standard technique, faces the challenge of studying the products with higher base pairs, need of sequencer, while PCR-RFLP although being easy to perform with less infrastructure and also cost effective, is not precise. There are even inherent limitations of comparing RFLP RE digestion banding patterns. Apart from visual inaccuracies, even the insufficient PCR amplification product and amplification errors may hinder the appropriate matching and in turn interpretation of relapse and reinfection based on RFLP pattern.

As far as selection of marker for genotyping is concerned, it is the highly polymorphic gene that is preferred. However, if the region to be studied is highly polymorphic then it leads to amplification problem due to the polymorphism existing in the primer binding region. We used MSP-3 gene family for RFLP genotyping on account of the polymorphism documented with this gene. However, recent research [35] also documents extensive recombination as well as gene conversion in this gene. This means,* P. vivax* genotype identified at the instance of recurrence, even though may belong to the original vivax genotype present at the time of first episode, might have undergone recombination leading to identification of different genotype at the time of recurrence classifying it as reinfection. However, for the current study we can assume this effect to be constant across the four groups compared making the final interpretations still valid.

Further studies in various other geographic areas are needed for taking decision on policy change, even if that has to be made.

In the present study large number of samples showed nonamplification for genotyping methods used. This was also observed in the study by Maestre et al. [36] where almost 35% nonamplifications occurred. The reasons of nonamplification could be varied viz; absence of or less amount of DNA in the sample, presence of PCR inhibitors in the isolated DNA, polymorphism present in the primer binding site itself (high diversity observed in the polymorphic regions studied). The PCR sequencing (MSP1) used in the study did involve different primer design for some of the nonamplifying samples (data not shown). Studying multiple polymorphic regions and their respective primers may yield more data (as seen in the present study, few samples not amplifying with MSP3*α*; showed amplification with MSP3*β* and vice a versa).

The study also determined the extent of polymorphism in* P. vivax* in Mumbai, India as a part of the PCR sequencing method employed for genotyping analysis. This was important, as the data regarding the sequence variations among* P. vivax* Indian field isolates is scanty. The results showed 52 genetic subtypes in a total of 143 isolates (paired Primary and recurrence samples) analyzed and a quite high PvMSP-1 polymorphism as analyzed by PCR sequencing method. The analysis and studying sequence of more than one polymorphic region would yield even more* P. vivax* sub-types. The simultaneous use of more than one genetic marker in this kind of study may enhance the knowledge of genetic diversity existing in the parasite populations. Nevertheless, high genetic diversity has been reported with the use of one MSP-1 gene and therefore, use of this marker is justified for the purposes of genotyping. The genetic diversity of* P. vivax* has also been demonstrated earlier in other geographic areas [37]. Understanding the extent of polymorphism in MSP-1 and the resulting genetic diversity in* P. vivax* populations could help in implementing malaria control activities, being a crucial step for the development of a malaria vaccine although the significance of the diverse polymorphisms and their prevalence is unknown.

From this study it is evident that, although the incidence of probable resistance to various PQ regimens, used in the present study, is fairly low at this point (relapse rate ranging from 1.47% to 4.58% using different methods of classification of recurrence), it is anticipated that resistance is likely to increase and therefore, continuous monitoring of the therapy with PQ is warranted. One should keep in mind the propagation of the resistant strain which may lead to increased relapse rate in near future. Also, this is an indication of declining efficacy of PQ which could be due to increasing resistance to the drug.

## 5. Limitations

There are several methodological limitations of the study. One of the major limitation of the study is the nonrandomization of the No PQ group due to ethical reasons which forced us to select the patients for this group based on those who did not receive PQ because of the clinical reasons such as Hg < 10 gm/dL; G6PD deficiency, pregnancy, lactation, and so forth. However, we do not believe these variables might have had any effect on the recurrence rate. Additionally, even though we used this group to compare with rest of the PQ regimens tested, the comparative number of recurrence within 3 PQ groups presented in the table are self-explanatory. The patients in this study were followed up only for 6 months. The number thus presented in the study may be an underestimation as the late relapses may not have been adequately captured. The longer follow-up period pose challenge of more drop outs on account of data lost to followup. Additionally, because this was a comparative study it can be assumed that the longer followup may not influence the comparison even with increase in the number of patients showing recurrence. The study used month of recurrence as one of the method of classifying recurrence as relapse or reinfection. We certainly understand that this method of classifications of recurrences is deeply problematic. While a case may be made for relapses during low-transmission season, no case at all may be made for classifying recurrences as reinfection during the transmission season. Even low probability transmission may suffice to seriously confound this classification system. However, typically a trend in increase in cases of malaria is noted during the monsoon season in Mumbai, the geographic area where the study was carried out. In view of this scenario this method was considered. Our objective of including this method in the current study was to basically compile the possible methods of differentiation and present an analysis of the data even though it may not be an accurate method.

We did not use body weight of the patients enrolled in the study. This variable might have helped in calculating per kg dose of the PQ that patients received. The low total PQ dose per kilogram of body weight [28, 30, 38] as well as genetic mutations in CYP2D6 [39] may influence the effective PQ dose available and low dose leading to relapse. However, as per WHO recommendations, per kg PQ dose is 0.25 mg base/kg body weight [40]. Considering the average body weight of Indian subjects ranging between 50 and 60 kg, the regimens used in the current study would yield the recommended per kg dose thus over ruling the confounding effect of the weight. Also, the prevalence of CYP2D6 polymorphism is reported to be as low as 3.36% [41]. Additionally higher PQ dose included in the present study and the recurrence and relapse rates observed in this group likely overcome the confounding effect of these factors. Several factors related to parasite variants, host factors and the epidemiological factors including vector control, transmission dynamics; the limitations of the existing methods to identify true relapses can influence and confound the analysis of true resistance to PQ. This indicates that in the absence of reliable genotyping methods, the recurrence monitoring can be a good surrogate marker to compare provided it is appropriate to assume the constant reinfection rate in the given geographical area where the study population is residing.

## 6. Conclusions

The study documented statistically significant higher recurrence rate in patients not given PQ as compared to patients receiving different PQ regimens. There was discordance in the methods (namely, month of recurrences, RFLP, or sequencing) used to differentiate relapse from reinfection. There could be several reasons for discordance. None of the methods are suitable given their limitation. The lack of concordance in the methods used has implications on the interpretation of the results and therefore warrants caution.

Using the available methods, the relapse rate ranging from 1.47% to 4.58% was noted in the patients receiving different PQ regimens. Considering that the paired samples classified as “reinfection” by RFLP or sequencing could be the cases of “relapse,” the probable resistance to PQ could be even higher indicating the decreased effect of PQ to eradicate liver forms of* P. vivax*, hypnozoites. This is applicable to the higher doses of PQ as well.

The study found that PQ regimens used to be safe without any major or unexpected adverse events, although statistically significantly higher adverse events were noted in the higher dose PQ (30 mg × 14 days) leading to significantly higher drop outs due to discontinuation of the treatment.

The study also documented lots of genetic diversity in the types of genotype identified using the PCR sequencing method of genotyping, indicating diversity of type of* P. vivax* strains prevalent in the population (geographic area) studied.

## Figures and Tables

**Table 1:** Baseline demographic characteristics of the patients enrolled in the study.

PQ regimens	Group A (no PQ)	Group B (15 mg/day × 14 days)	Group C (30 mg/day × 7 days)	Group D (30 mg/day × 14 days)
Number of patients enrolled	397	398	381	380
Total drop outs	92 (23.1%)	76 (19%)	83 (21.7%)	63 (16.5%)
Number of patients who completed 6 months of followup	305	322	298	317
Age in years (mean ± SD)	18–76 (30 ± 14)	18–76 (31 ± 12)	18–74 (32 ± 13)	18–70 (32 ± 11)
Gender				
Male	374	378	364	365
Female	23	20	17	15
Parasitemia	120–16780/*μ*L	160–17600/*µ*L	175–15620/*μ*L	105–19239/*μ*L

**Table 2:** Differentiation of recurrences as relapse or reinfection.

PQ regimens	Group A (no PQ)	Group B (15 mg/day × 14 days)	Group C (30 mg/day × 7 days)	Group D (30 mg/day × 14 days)
Number of patients who completed 6 months of followup	305	322	298	317
Number of patients showing recurrence (%)∗	50 (16.39)	26 (8.07)	30 (10.07)	21 (6.62)

As per the month of recurrence				
Number of relapses	21	5	12	9
Number of reinfections	29	21	18	12
Relapse rate∗∗	**6.89%**	**1.55%**	**4.00%**	**2.84%**
Reinfection rate	**9.51%**	**6.52%**	**6.00%**	**3.79%**

Genotyping (PCR-RFLP)				
Paired primary and recurrence samples available for analysis	24	15	18	16
Number of PCR-RFLP genotyping results available for the paired samples	16	11	11	9
Number of samples showing same genotypes	8	3	5	5
Number of samples showing different genotypes	8	8	6	4
Relapse rate∗∗∗	**8.20%**	**2%**	**4.58%**	**3.68%**
Reinfection rate	**8.20%**	**5.82%**	**5.49%**	**2.9%**

Genotyping (PCR sequencing)				
Paired primary and recurrence samples available and analyzed	24	15	18	16
Number of PCR sequencing results available for the paired samples	12	11	13	13
Number of samples showing same genotypes	2	2	2	3
Number of samples showing different genotypes	10	9	11	10
Relapse rate	**2.73%**	**1.47%**	**1.55%**	**1.53%**
Reinfection rate	**13.66%**	**6.60%**	**8.52%**	**5.09%**

*P = 0.004, **P = 0.009, ***P = 0.007.

**Table 3:** AEs of CQ and PQ in the four groups.

PQ regimens	Group A (no PQ)	Group B (15 mg/day × 14 days)	Group C (30 mg/day × 7 days)	Group D (30 mg/day × 14 days)
Number of patients enrolled	397	398	381	380
AEs of CQ∗	1	0	4	4
Drop outs (discontinuation of CQ) due to CQ AEs	0	0	0	1
AEs of PQ∗∗	3	5	10	13
Drop outs (discontinuation of PQ) due to PQ AEs∗∗∗	0	2	2	7
Total drop outs due to AEs (CQ and PQ)∗∗∗∗	None	2	2	8

*P = 0.033, **P = 0.006, ***P = 0.003, ****P = 0.001.

**Table 4:** Comparison of the methods for their concordance to classify the cases of recurrence as relapse of reinfection.

All 3 methods	RFLP and PCR sequencing	Month of recurrence and PCR-RFLP	Month of recurrence and PCR sequencing
15/33 (45%)	19/33 (57.58%)	16/33 (48.48%)	5/33 (15.15%)
